# Application and interpretation of machine learning models in predicting the risk of severe obstructive sleep apnea in adults

**DOI:** 10.1186/s12911-023-02331-z

**Published:** 2023-10-19

**Authors:** Yewen Shi, Yitong Zhang, Zine Cao, Lina Ma, Yuqi Yuan, Xiaoxin Niu, Yonglong Su, Yushan Xie, Xi Chen, Liang Xing, Xinhong Hei, Haiqin Liu, Shinan Wu, Wenle Li, Xiaoyong Ren

**Affiliations:** 1 Department of Otorhinolaryngology Head and Neck Surgery, The Second Affiliated Hospital of Xi’an Jiaotong University, NO. 157 Xi Wu Road, Xi’an, Shaan’xi Province China; 2 School of Computer Science and Engineering, Xi’an University of Technology, Xi’an, Shaan’xi Province China; 3 School of Medicine, Eye Institute of Xiamen University, Xiamen University, Xiamen, Fujian Province China; 4 Molecular Imaging and Translational Medicine Research Center, State Key Laboratory of Molecular Vaccinology and Molecular Diagnostics, Xiamen University, Xiamen, Fujian Province China

**Keywords:** Obstructive sleep apnea, Prediction model, Machine learning, Risk factor, Shapley additive explanations, Gradient boosting machine

## Abstract

**Background:**

Obstructive sleep apnea (OSA) is a globally prevalent disease with a complex diagnostic method. Severe OSA is associated with multi-system dysfunction. We aimed to develop an interpretable machine learning (ML) model for predicting the risk of severe OSA and analyzing the risk factors based on clinical characteristics and questionnaires.

**Methods:**

This was a retrospective study comprising 1656 subjects who presented and underwent polysomnography (PSG) between 2018 and 2021. A total of 23 variables were included, and after univariate analysis, 15 variables were selected for further preprocessing. Six types of classification models were used to evaluate the ability to predict severe OSA, namely logistic regression (LR), gradient boosting machine (GBM), extreme gradient boosting (XGBoost), adaptive boosting (AdaBoost), bootstrapped aggregating (Bagging), and multilayer perceptron (MLP). All models used the area under the receiver operating characteristic curve (AUC) was calculated as the performance metric. We also drew SHapley Additive exPlanations (SHAP) plots to interpret predictive results and to analyze the relative importance of risk factors. An online calculator was developed to estimate the risk of severe OSA in individuals.

**Results:**

Among the enrolled subjects, 61.47% (1018/1656) were diagnosed with severe OSA. Multivariate LR analysis showed that 10 of 23 variables were independent risk factors for severe OSA. The GBM model showed the best performance (AUC = 0.857, accuracy = 0.766, sensitivity = 0.798, specificity = 0.734). An online calculator was developed to estimate the risk of severe OSA based on the GBM model. Finally, waist circumference, neck circumference, the Epworth Sleepiness Scale, age, and the Berlin questionnaire were revealed by the SHAP plot as the top five critical variables contributing to the diagnosis of severe OSA. Additionally, two typical cases were analyzed to interpret the contribution of each variable to the outcome prediction in a single patient.

**Conclusions:**

We established six risk prediction models for severe OSA using ML algorithms. Among them, the GBM model performed best. The model facilitates individualized assessment and further clinical strategies for patients with suspected severe OSA. This will help to identify patients with severe OSA as early as possible and ensure their timely treatment.

**Trial registration:**

Retrospectively registered.

## Background

Obstructive sleep apnea (OSA) refers to apnea and hypoventilation caused by repeated collapse and obstruction of the upper airway during sleep. OSA leads to frequent hypoxemia, hypercapnia, and sleep architecture disorders [1]. Epidemiological studies have shown that OSA has a high prevalence in adults, with approximately one billion people worldwide suffering from OSA, and the number of cases of moderate to severe OSA, for which treatment is generally recommended, is estimated to be almost 425 million [2]. As OSA progresses, the patient develops symptoms such as daytime sleepiness, hypomnesia, and inattention, which reduce their quality of life, impair work performance, and result in a higher risk of car accidents [3]. Moreover, severe OSA could cause dysfunction of multiple organ systems, such as the cardiovascular, endocrine, and nervous systems [4]. Due to the increasing prevalence and mortality of OSA and related complications [5], timely detection and treatment are crucial, especially in patients with severe OSA.

Because of the lack of symptom specificity and the complexity of diagnostic methods, OSA diagnosis remains difficult despite its high prevalence. One study showed that approximately 80% of patients with clinical symptoms of OSA were not diagnosed promptly [2]. Polysomnography (PSG) is the gold standard for OSA diagnosis [6]. However, PSG has many limitations: (i) The test is not widely available, waiting times are long, and the test takes a substantial amount of time. (ii) PSG technology is complex and specialized, and the obtained data are numerous and highly variable, so data interpretation is affected by personal factors and errors. (iii) The monitoring cost is high, and the economic burden is heavy. Therefore, it is crucial to develop a more convenient and accurate method to detect OSA and determine its severity.

Previous studies used various physiological data to develop predictive models of OSA to compensate for the defects of PSG, including clinical information [7–13], electrocardiograms [14–16], electroencephalograms [17–19], and oxyhemoglobin saturation [20–22]. However, the acquisition of partial physiological information requires evaluation by professional physicians and specialized equipment. In this respect, it does not minimize the preliminary screening process. Therefore, information that can be self-reported by patients is the most readily available and more suitable for inclusion in predictive models. Many parameters that are associated with the occurrence and severity of OSA have been identified in previous studies. The most well-recognized parameters are gender, age, body mass index (BMI), waist circumference, neck circumference, unhealthy lifestyle habits, and symptoms suggestive of OSA [23–26]. However, there is currently a lack of methods that use these parameters to rapidly and robustly diagnose OSA and determine its severity.

Machine learning (ML), a type of artificial intelligence, has been widely used in medical data analysis in recent years [27]. ML can be employed to detect complex relationships between predictors and outcomes and to improve the accuracy of analysis through continuous online learning. Because of its high efficiency, low cost, and convenience, ML has become a popular method for disease risk prediction and preliminary screening [28], including sleep medicine [29, 30]. ML shows the potential to overcome the challenges in OSA diagnosis.

Therefore, in the present study, we propose an interpretable ML model for predicting the risk of severe OSA in adults and analyzing the risk factors. The determination of OSA severity is important in early screening in a clinical context. This model provides individual strategies for severe OSA patients and aids in timely diagnosis and treatment.

## Methods

### Study population

This was a retrospective study. All patients (*n* = 1656) presented at the Second Affiliated Hospital of Xi’an Jiaotong University, China, between 2018 and 2021. Inclusion criteria were as follows: (i) age > 18 years old; (ii) with symptoms suggestive of OSA and related complications such as snoring, breathing cessations during sleep, and hypertension; and (iii) willingness to undergo PSG. Subjects were excluded (i) if they had craniofacial abnormalities/disorders; (ii) if they had neuromuscular disorders; (iii) if they were diagnosed with central sleep apnea syndrome based on PSG; (iv) if they had previously received sleep treatment; or (v) in case of long-term usage of medications known to affect sleep. This study was approved by the Ethics Committee of the Second Affiliated Hospital of Xi’an Jiaotong University. All participants provided informed consent for data collection and analysis. All data were anonymized.

### Data collection and feature selection

All subjects underwent PSG in a temperature-controlled and sound-attenuated room, supervised by the night-shift staff. Two experienced sleep physicians scored the PSG findings according to the American Academy of Sleep Medicine (AASM) criteria [31]. The apnea-hypopnea index (AHI) was defined as the total number of apneas and hypopneas per hour of sleep. OSA was defined as AHI ≥ 5 and severe OSA was defined as AHI ≥ 30.

A literature search was conducted to identify collected variables, including demographic data, lifestyle habits, medical history, symptoms suggestive of OSA, and sleep questionnaires. Demographic data included gender, age, BMI, neck circumference, and waist circumference. Lifestyle habits included smoking, drinking, lack of exercise, poor sleep, emotional instability, and stress. Hypertension, family history of hypertension, heart disease (self-reported arrhythmia, angina, coronary artery disease, or heart failure), diabetes, and hypothyroidism were self-reported medical history questions. Symptoms suggestive of OSA included snoring, breathing cessations during sleep, hypomnesia, and inattention. Sleep questionnaires included the Epworth Sleepiness Scale (ESS) [32], the Berlin questionnaire (BQ) [33], and the STOP-BANG questionnaire (SBQ) [34].

By comparing the clinical characteristics of the severe and non-severe OSA groups, the risk factors for predicting severe OSA were analyzed using the univariate analysis, and they were incorporated into ML models as characteristic variables. Additionally, the multivariate logistic regression analysis were also used to obtain independent predictors associated with severe OSA.

### Machine learning models

Six advanced ML algorithms were used to detect severe OSA, namely adaptive boosting (AdaBoost) [35], logistic regression (LR) [36], multilayer perceptron (MLP) [37], bootstrapped aggregating (Bagging) [38], gradient boosting machine (GBM) [39], and extreme gradient boost (XGBoost) [40].

Subjects were randomly divided into a training set and a test set with a ratio of 7:3. The training set was used to establish the prediction models. Each model was trained using 10-fold cross-validation, where each repetition was used as the test to overcome sampling bias. And the models were applied and validated in the test set.

The grid search and internal cross-validation were used to find the best hyperparameters of models in the training set (Table 1). Further analysis was performed using Python v3.10.9 with the pandas v1.5.3, streamlit v1.22.0, numpy v1.23.5, imblearn v0.10.1, matplotlib v3.7.0, sklearn v1.2.1, xgboost v1.7.5, shap v0.41.0, seaborn v0.12.2 packages (the source code is available on https://github.com/Wu-Shi-Nan/SOSA).

**Table 1:** The hyperparameters for proposed six machine learning models

Model	Hyperparameters
AdaBoost	n_estimators:10
	learning_rate:1.0
	algorithm: SAMME.R
	base_estimator: deprecated
LR	penalty:none
	dual:False
	tol:1e-4
	c:1.0
	fit_intercept: True
	intercept_scaling:1.0
	class_weight: None
	solver:lbfgs
	max_iter:100
	verbose:0
	warm_start: False
	n_jobs: None
MLP	hidden_layer_sizes:100
	activation: relu
	solver: lbfgs
	alpha:0.0001
	learning_rate: constant
	learning_rate_init:0.01
	power_t:0.5
	max_iter:200
	shuffle: True
Bagging	n_estimators:10
	bootstrap: True
	bootstrap_features: False
	oob_score: False
	warm_start: False
	n_jobs: None
	verbose:0
	base_estimator: deprecated
	max_samples:0.5
	max_features:0.5
GBM	n_estimators:100
	learning_rate:1.0
	max_depth:1.0
	subsample:1.0
	criterion: friedman_mse
	min_samples_split:2
	min_samples_leaf:1
	min_weight_fraction_leaf:0.0
	min_impurity_decrease:0.0
	init: None
	max_features: None
	verbose:0
	max_leaf_nodes: None
	warm_start: False
	validation_fraction:0.1
	n_iter_no_change: None
	tol:1e-4
	ccp_alpha:0.0
XGBoost	n_estimators:360
	max_depth:1
	learning_rate:1.6

AdaBoost, adaptive boosting; LR, logistic regression; Bagging, bootstrapped aggregating; MLP, multilayer perceptron; GBM, gradient boosting machine; and XGBoost, extreme gradient boost

### Statistical analysis

**Fig. 1:**
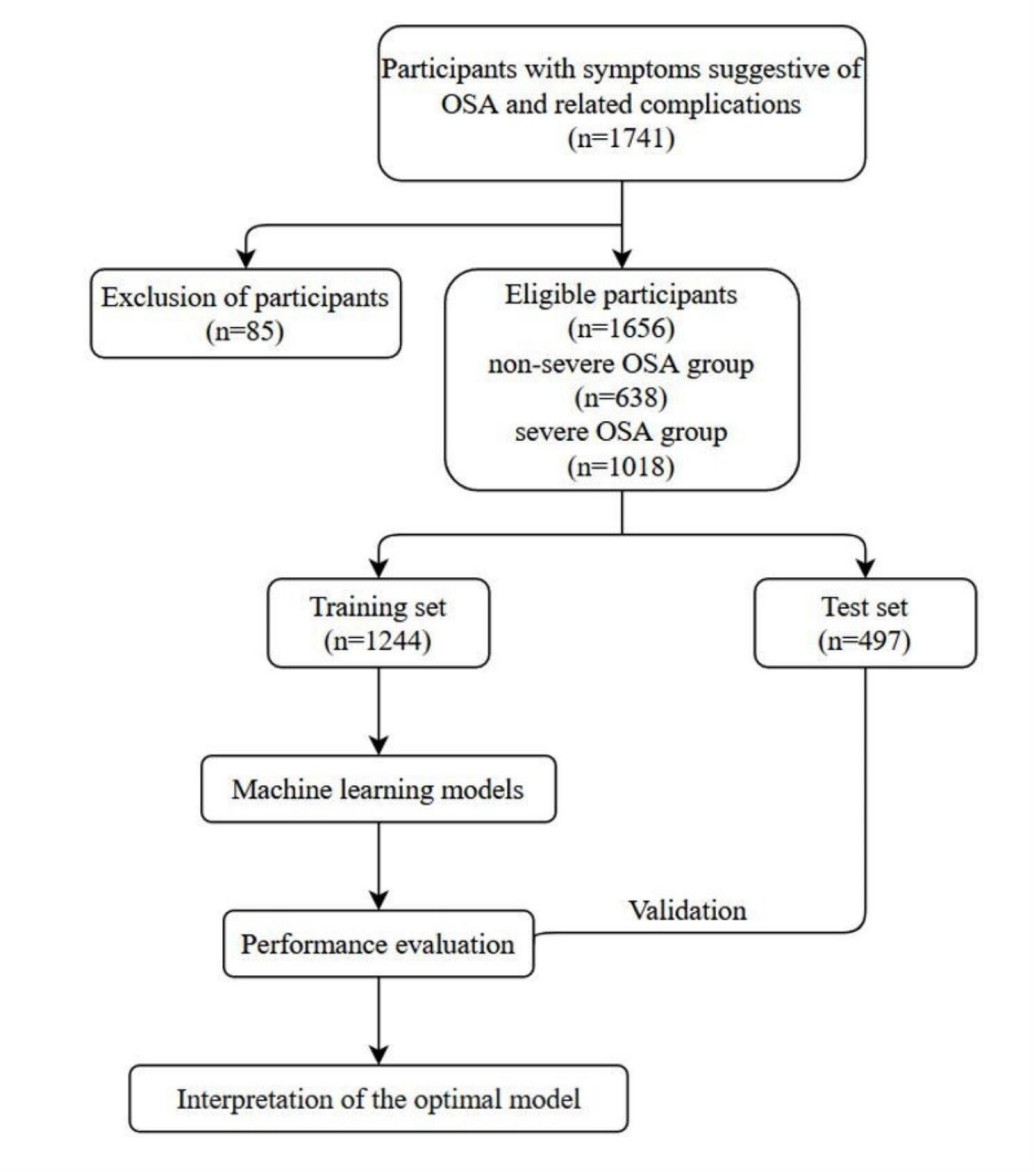
Schematic overview of the proposed method OSA, obstructive sleep apnea

All analyses were performed using R software (version 3.6.0). ML models and web calculators were built using Python (version 3.8). Continuous variables are presented as the median with interquartile range (IQR). Categorical variables are presented as numbers with proportions. Differences between groups were compared by the Wilcoxon rank-sum test, the Chi-squared test, or Fisher’s exact test. Univariate and multivariate analyses were conducted to analyze the risk factors for predicting severe OSA. *P* < 0.05 was set as the threshold for statistical significance. The prediction performance of the models was evaluated based on the area under the receiver operating characteristic curve (AUC), accuracy, sensitivity, and specificity. SHapley Additive exPlanations (SHAP) plots were drawn to interpret predictive results and to analyze the relative importance of risk factors. An online calculator based on the model with the best AUC value was capable of estimating the risk of severe OSA in individuals. The proposed approach is shown in Fig. 1.

## Results

### Comparisons between patients with and without severe OSA

According to the PSG results, 1656 enrolled OSA patients were divided into the non-severe OSA group (AHI < 30 per hour) and the severe OSA group (AHI ≥ 30 per hour).

The differences between the two groups in terms of gender, age, BMI, waist circumference, neck circumference, smoking, drinking, lack of exercise, hypertension, snoring, breathing cessations during sleep, hypomnesia, ESS, BQ, and SBQ (hypomnesia *P* < 0.05, other *P* < 0.001) were statistically significant. Compared with the non-severe OSA patients, the severe OSA patients were more likely to be male, to be older, to have a higher BMI, to have a larger waist circumference, to have a larger neck circumference, to smoke, to drink, and to not do exercise. Severe OSA patients had more pronounced symptoms of hypertension, snoring, breathing cessations during sleep, and hypomnesia, and their sleep questionnaire results were more serious (Table 2).

**Table 2:** Comparisons of variables between patients with and without severe OSA

Variables	Total (n = 1656)	Non severe OSA (n = 638)	Severe OSA (n = 1018)		*P* value
**Demographic data**					
Gender [n (%)]					< 0.001
Female	298 (18)	182 (29)		116 (11)	
Male	1358 (82)	456 (71)		902 (89)	
Age (years) [M (Q1, Q3)]	40 (33, 50)	38 (30, 50)		41 (34, 50)	< 0.001
BMI (kg/m^2^) [M (Q1, Q3)]	26.52 (24.33, 29.07)	25.02 (23.1, 27.34)		27.68 (25.44, 29.98)	< 0.001
Waist circumference(cm) [M (Q1, Q3)]	97 (91, 104)	93 (86, 99)		100 (95, 106)	< 0.001
Neck circumference(cm) [M (Q1, Q3)]	39 (37, 41)	38 (35, 40)		40 (38, 42)	< 0.001
**Lifestyle habits**					
Smoking [n (%)]					< 0.001
0	966 (58)	468 (73)		498 (49)	
1	690 (42)	170 (27)		520 (51)	
Drinking [n (%)]					< 0.001
0	1043 (63)	472 (74)		571 (56)	
1	613 (37)	166 (26)		447 (44)	
Lack of exercise [n (%)]					< 0.001
0	656 (40)	287 (45)		369 (36)	
1	1000 (60)	351 (55)		649 (64)	
Poor sleep [n (%)]					0.976
0	896 (54)	346 (54)		550 (54)	
1	760 (46)	292 (46)		468 (46)	
Emotional instability [n (%)]					0.472
0	1186 (72)	450 (71)		736 (72)	
1	470 (28)	188 (29)		282 (28)	
Stress [n (%)]					0.904
0	1211 (73)	465 (73)		746 (73)	
1	445 (27)	173 (27)		272 (27)	
**Medical history**					
Hypertension [n (%)]					< 0.001
0	1226 (74)	530 (83)		696 (68)	
1	430 (26)	108 (17)		322 (32)	
Family history of hypertension [n (%)]					0.458
0	1157 (70)	453 (71)		704 (69)	
1	499 (30)	185 (29)		314 (31)	
Heart disease [n (%)]					0.715
0	1535 (93)	589 (92)		946 (93)	
1	121 (7)	49 (8)		72 (7)	
Diabetes [n (%)]					0.384
0	1586 (96)	615 (96)		971 (95)	
1	70 (4)	23 (4)		47 (5)	
Hypothyroidism [n (%)]					0.310
0	1616 (98)	619 (97)		997 (98)	
1	40 (2)	19 (3)		21 (2)	
**OSA related symptoms**					
Snoring [n (%)]					< 0.001
0	39 (2)	36 (6)		3 (0)	
1	1617 (98)	602 (94)		1015 (100)	
Breathing cessations during sleep [n (%)]					< 0.001
0	312 (19)	194 (30)		118 (12)	
1	1344 (81)	444 (70)		900 (88)	
Hypomnesia [n (%)]					0.020
0	626 (38)	264 (41)		362 (36)	
1	1030 (62)	374 (59)		656 (64)	
Inattention [n (%)]					0.773
0	770 (46)	300 (47)		470 (46)	
1	886 (54)	338 (53)		548 (54)	
**Sleep questionnaires**					
ESS [n (%)]					< 0.001
None	571 (34)	300 (47)		271 (27)	
Slight	530 (32)	211 (33)		319 (31)	
Moderate	325 (20)	91 (14)		234 (23)	
High	230 (14)	36 (6)		194 (19)	
BQ [n (%)]					< 0.001
Low risk	143 (9)	120 (19)		23 (2)	
High risk	1513 (91)	518 (81)		995 (98)	
SBQ [n (%)]					< 0.001
Low risk	225 (14)	183 (29)		42 (4)	
High risk	1431 (86)	455 (71)		976 (96)	

**OSA**, obstructive sleep apnea; **BMI**, body mass index; **ESS**, Epworth Sleepiness Scale; **BQ**, Berlin questionnaire; and **SBQ**, STOP-BANG questionnaire

### Univariate and multivariate logistic regression analyses

A total of 1159 patients were assigned to the training set (70% of the total population). In the training set, univariate analysis revealed that male sex, age, BMI, waist circumference, neck circumference, smoking, drinking, lack of exercise, hypertension, snoring, breathing cessations during sleep, hypomnesia, ESS, BQ, and SBQ (hypomnesia *P* < 0.05, other *P* < 0.001) were significantly associated with severe OSA. All these parameters were included in multivariate LR analysis. The results showed that male sex (OR: 1.509, 95%CI: 1.014–2.245, *P* < 0.05), age (OR: 1.017, 95%CI: 1.005–1.029, *P* < 0.05), BMI (OR: 1.073, 95%CI: 1.005–1.146, *P* < 0.005), waist circumference (OR: 1.045, 95%CI: 1.020–1.072, *P* < 0.001), smoking (OR: 1.539, 95%CI: 1.172–2.020, *P* < 0.05), snoring (OR: 5.859, 95%CI: 1.597–21.418, *P* < 0.05), breathing cessations during sleep (OR: 1.656, 95%CI: 1.206–2.273, *P* < 0.05), moderate ESS (OR: 1.956, 95%CI: 1.389–2.754, *P* < 0.001), high ESS (OR: 2.692, 95%CI: 1.738–4.170, *P* < 0.001), BQ (OR: 3.782, 95%CI: 2.217–6.452, *P* < 0.001), and SBQ (OR: 2.108, 95%CI: 1.379–3.223, *P* = 0.001) were independent predictors of severe OSA (Table 3).

**Table 3:** Univariate and multivariate logistic regression analyses

Variables	Univariate analysis		Multivariate analysis	
	OR (95% CI)	*P* value	OR (95% CI)	*P* value
Gender				
Female	Ref	Ref	Ref	Ref
Male	3.104 (2.396–4.020)	< 0.001	1.509 (1.014–2.245)	0.042
Age	1.016 (1.008–1.025)	< 0.001	1.017 (1.005–1.029)	0.004
BMI	1.266 (1.223–1.310)	< 0.001	1.073 (1.005–1.146)	0.034
Waist circumference	1.099 (1.085–1.113)	< 0.001	1.045 (1.020–1.072)	< 0.001
Neck circumference	1.252 (1.212–1.295)	< 0.001	0.998 (0.940–1.060)	0.952
Smoking				
0	Ref	Ref	Ref	Ref
1	2.875 (2.320–3.561)	< 0.001	1.539 (1.172–2.020)	0.002
Drinking				
0	Ref	Ref	Ref	Ref
1	2.226 (1.794–2.762)	< 0.001	1.149 (0.874–1.511)	0.319
Lack of exercise				
0	Ref	Ref	Ref	Ref
1	1.438 (1.175–1.759)	< 0.001	1.037 (0.805–1.336)	0.779
Poor sleep				
0	Ref	Ref	Ref	Ref
1	1.008 (0.827–1.230)	0.935	/	/
Emotional instability				
0	Ref	Ref	Ref	Ref
1	0.917 (0.737–1.141)	0.438	/	/
Stress				
0	Ref	Ref	Ref	Ref
1	0.980(0.784–1.225)	0.859	/	/
Hypertension				
0	Ref	Ref	Ref	Ref
1	2.270 (1.776–2.902)	< 0.001	1.207 (0.887–1.643)	0.231
Family history of hypertension				
0	Ref	Ref	Ref	Ref
1	1.092 (0.879–1.356)	0.425	/	/
Heart disease				
0	Ref	Ref	Ref	Ref
1	0.915 (0.627–1.334)	0.644	/	/
Diabetes				
0	Ref	Ref	Ref	Ref
1	1.294 (0.778–2.153)	0.320	/	/
Hypothyroidism				
0	Ref	Ref	Ref	Ref
1	0.686 (0.366–1.287)	0.240	/	/
Snoring				
0	Ref	Ref	Ref	Ref
1	20.233 (6.209–65.927)	< 0.001	5.849 (1.597–21.418)	0.008
Breathing cessations during sleep				
0	Ref	Ref	Ref	Ref
1	3.333 (2.581–4.303)	< 0.001	1.656 (1.206–2.273)	0.002
Hypomnesia				
0	Ref	Ref	Ref	Ref
1	1.279 (1.044–1.567)	0.018	0.917 (0.713–1.179)	0.497
Inattention				
0	Ref	Ref	Ref	Ref
1	1.035 (0.849–1.262)	0.735	/	/
ESS				
None	Ref	Ref	Ref	Ref
Slight	1.674 (1.318–2.126)	< 0.001	1.237 (0.937–1.634)	0.133
Moderate	2.847 (2.124–3.814)	< 0.001	1.956 (1.389–2.754)	< 0.001
High	5.966 (4.032–8.827)	< 0.001	2.692 (1.738–4.170)	< 0.001
BQ				
Low risk	Ref	Ref	Ref	Ref
High risk	10.022 (6.335–15.853)	< 0.001	3.782 (2.217–6.452)	< 0.001
SBQ				
Low risk	Ref	Ref	Ref	Ref
High risk	9.346 (6.564–13.307)	< 0.001	2.108 (1.379–3.223)	0.001

**BMI**, body mass index; **ESS**, Epworth Sleepiness Scale; **BQ**, Berlin questionnaire; and **SBQ**, STOP-BANG questionnaire

### Performance of machine learning models

**Fig. 2:**
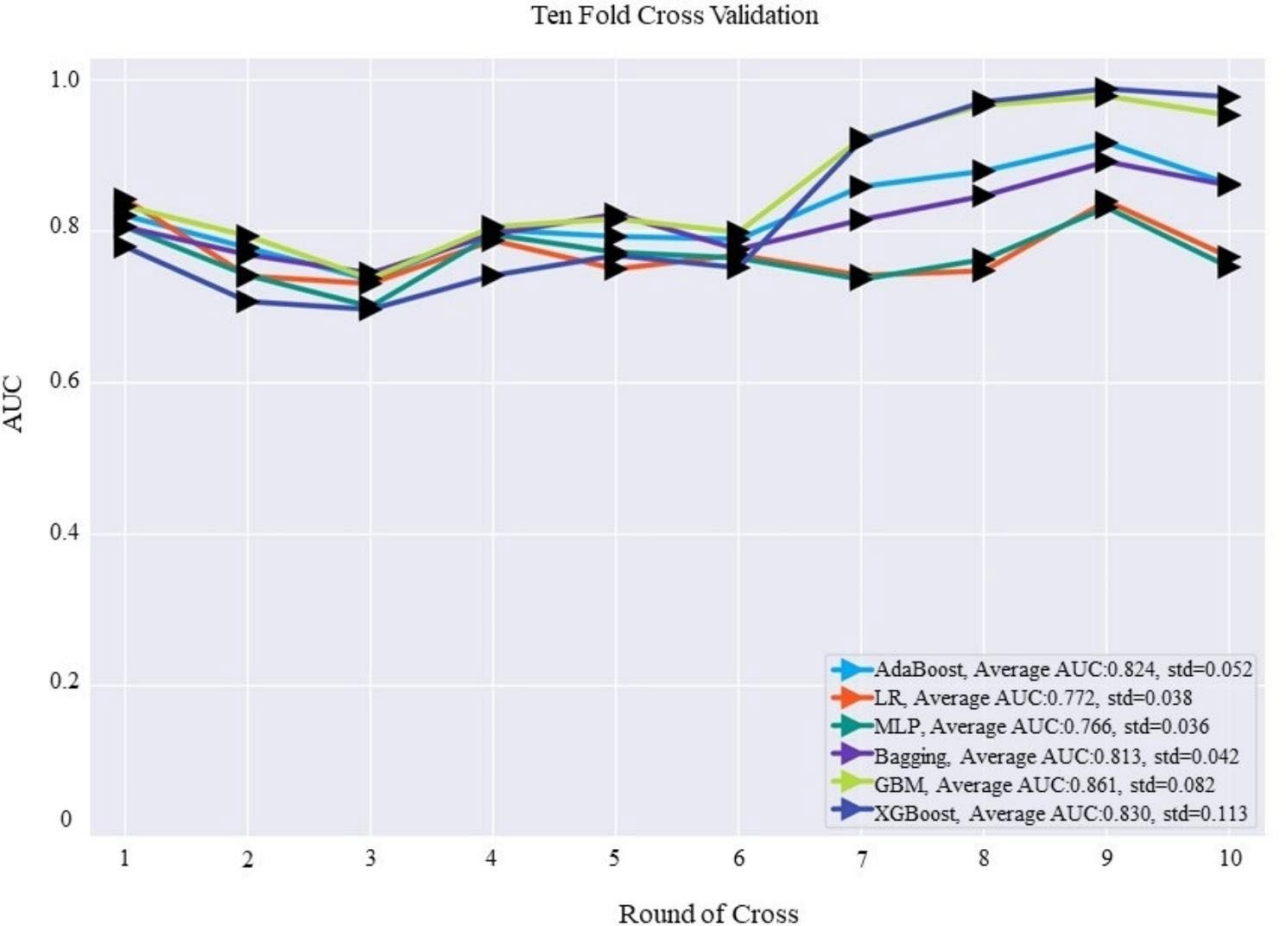
Ten-fold cross-validation of six ML models on the training group AUC, area under the receiver operating characteristic curve; AdaBoost, adaptive boosting; LR, logistic regression; Bagging, bootstrapped aggregating; MLP, multilayer perceptron; GBM, gradient boosting machine; and XGBoost, extreme gradient boost

**Fig. 3:**
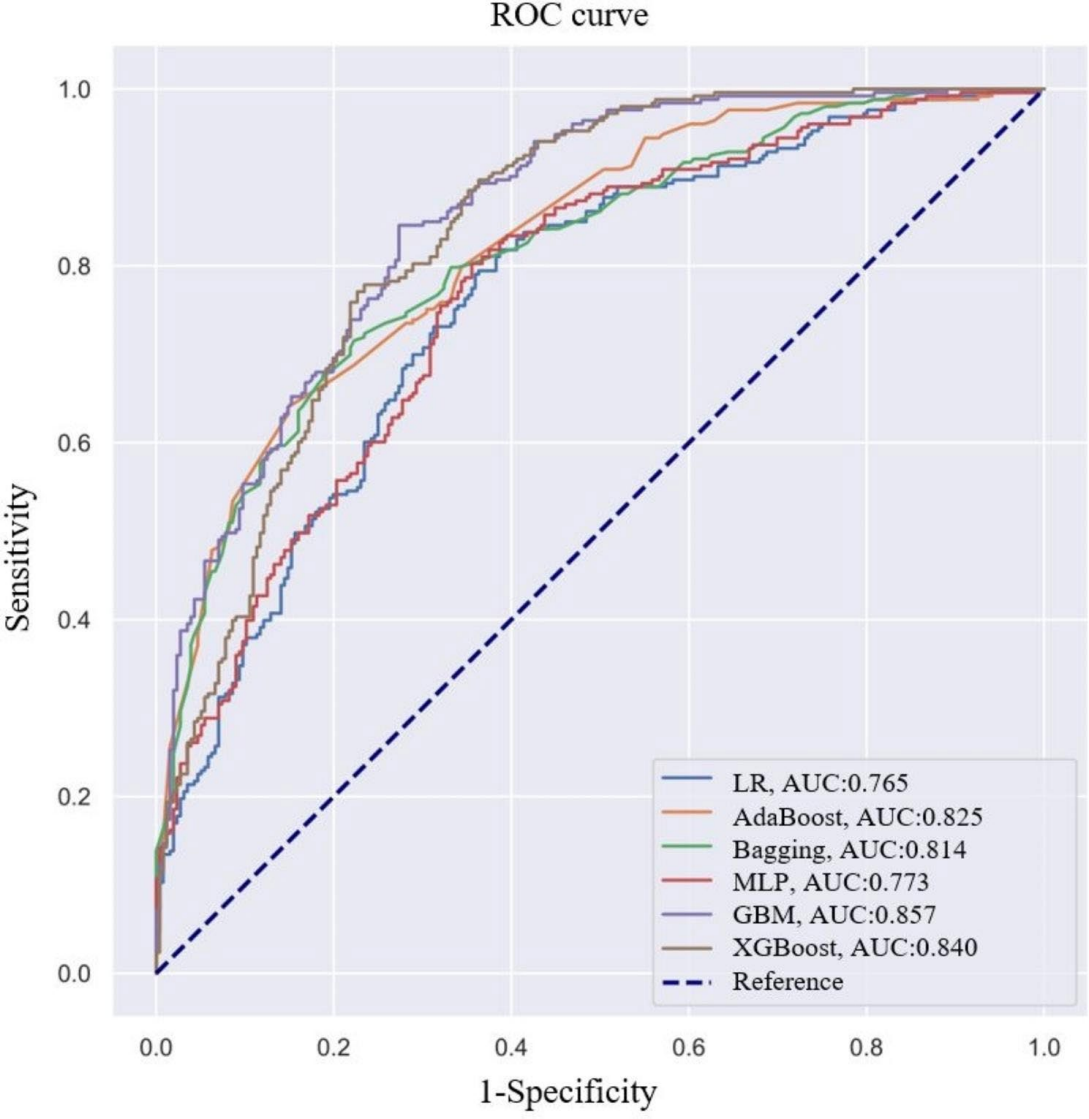
ROC curves of six ML models on the validation group ROC, receiver operating characteristic; AUC, area under the receiver operating characteristic curve; AdaBoost, adaptive boosting; LR, logistic regression; Bagging, bootstrapped aggregating; MLP, multilayer perceptron; GBM, gradient boosting machine; and XGBoost, extreme gradient boost

We designed six classification models and evaluated their ability to predict severe OSA. Each model was trained by 10-fold cross-validation on the training group. The GBM model had the highest average accuracy with an AUC of 0.861 (Fig. 2). The results of the validation group also showed that the AUC values of the six ML models ranged from 0.765 to 0.857, while the GBM model showed the best performance (Fig. 3). The proposed GBM model had an accuracy, sensitivity, and specificity of 0.766, 0.798, and 0.734, respectively (Table 4). Therefore, we chose the GBM model as the best prediction model for conducting further analyses.

**Table 4:** Performance of six ML models

Model	AUC	Accuracy	Sensitivity	Specificity
AdaBoost	0.825	0.723	0.751	0.695
LR	0.765	0.713	0.794	0.633
Bagging	0.814	0.747	0.715	0.727
MLP	0.773	0.717	0.802	0.633
GBM	0.857	0.766	0.798	0.734
XGBoost	0.840	0.752	0.771	0.713

**AUC**, area under the receiver operating characteristic curve; **AdaBoost**, adaptive boosting; **LR**, logistic regression; **Bagging**, bootstrapped aggregating; **MLP**, multilayer perceptron; **GBM**, gradient boosting machine; and **XGBoost**, extreme gradient boost

### Relative importance of variables in the GBM model

**Fig. 4:**
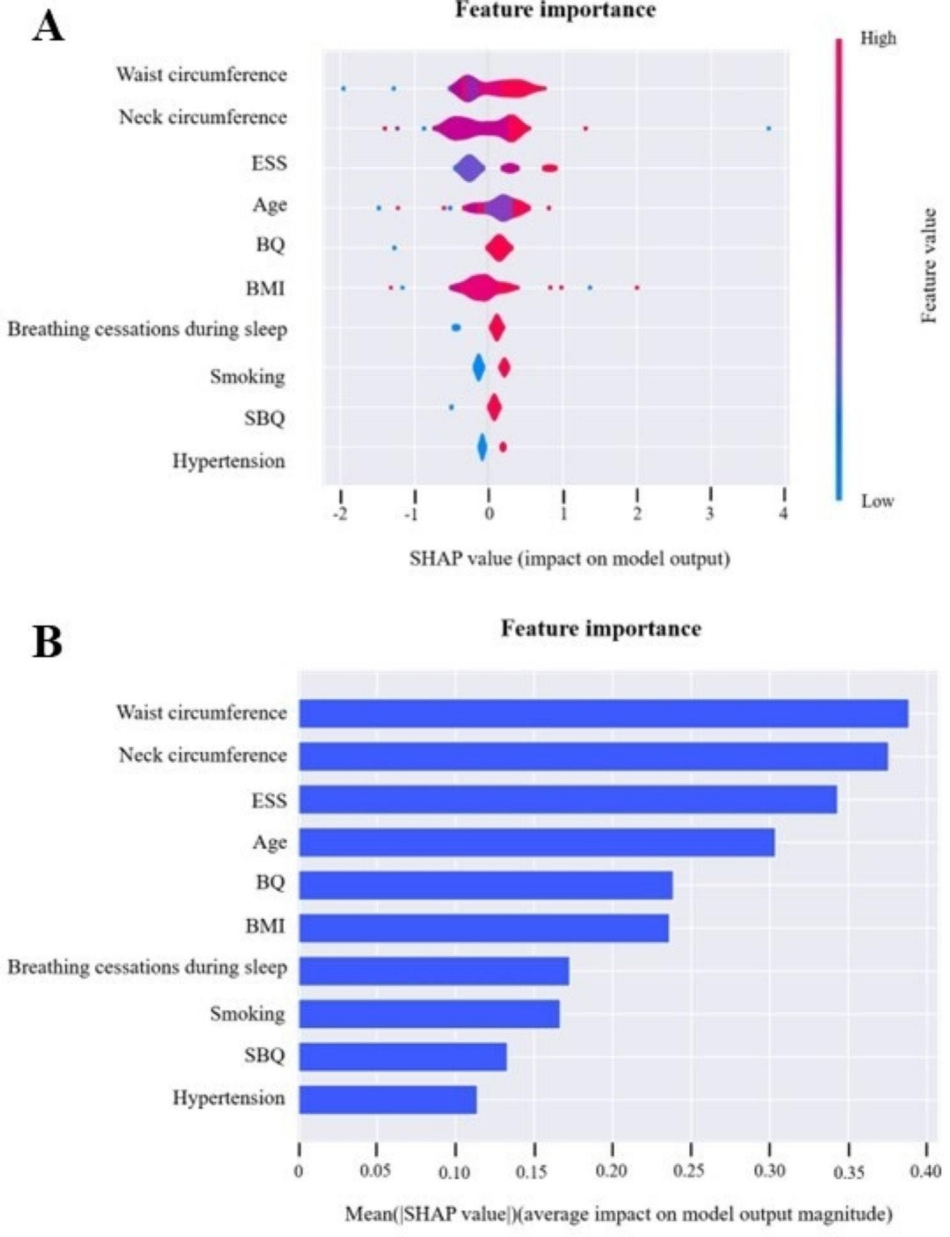
SHAP summary plot to illustrate the model predicting severe OSA at the feature level The features were ranked according to the sum of the SHAP values for all patients, and the SHAP values are used to show the distribution of the effect of each feature on the GBM model outputs. Each dot represents a case in the dataset. The color of a dot indicates the value of the feature, with blue indicating low values and red indicating high values. Only the top 10 important predictors are shown in the plot. SHAP, SHapley Additive exPlanations; ESS, Epworth Sleepiness Scale; BQ, Berlin questionnaire; BMI, body mass index; and SBQ, STOP-BANG questionnaire

To further analyze the relative importance of risk factors, we drew a SHAP summary plot. The importance of the 10 features that have the largest effect on the model output was explained. Waist circumference, neck circumference, ESS, age, and BQ were found to be the top five critical variables contributing to the diagnosis of severe OSA (Fig. 4).

### Interpretation of the GBM Model at the individual level

**Fig. 5:**
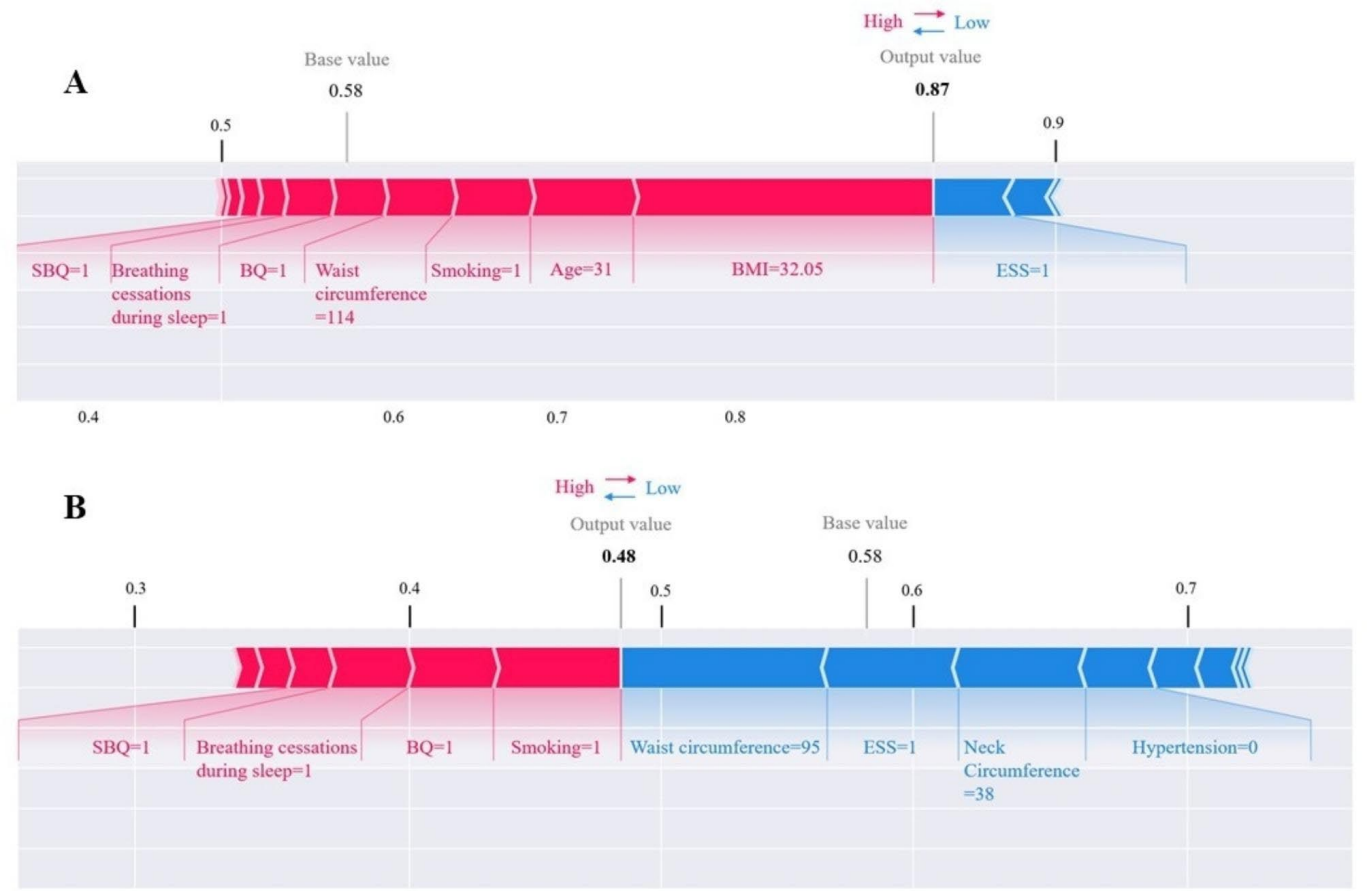
SHAP force plot to illustrate the model predicting severe OSA at the individual level Red and blue bars, respectively, represent the positive and negative effects of each predictor contributing to the occurrence of the outcome. The extent of the impact is represented by the size of the bar. ESS, Epworth Sleepiness Scale; BQ, Berlin questionnaire; BMI, body mass index; and SBQ, STOP-BANG questionnaire

The SHAP force plot was used to illustrate individual risk profiles. Two typical cases were analyzed to interpret the contribution of each variable to the predicted outcome in a single patient (Fig. 5). In Fig. 5A, the predicted probability for severe OSA was relatively high (0.87) due to many conditions, including high BMI (32.05 kg/m^2^) and waist circumference (114 cm), age (31 years), smoking, breathing cessations during sleep, and high SBQ and BQ. In contrast, in Fig. 5B, the probability of severe OSA was low (0.48) due to many conditions, including low waist circumference (95 cm) and neck circumference (38 cm), the absence of hypertension, and low ESS.

### Online calculator

**Fig. 6:**
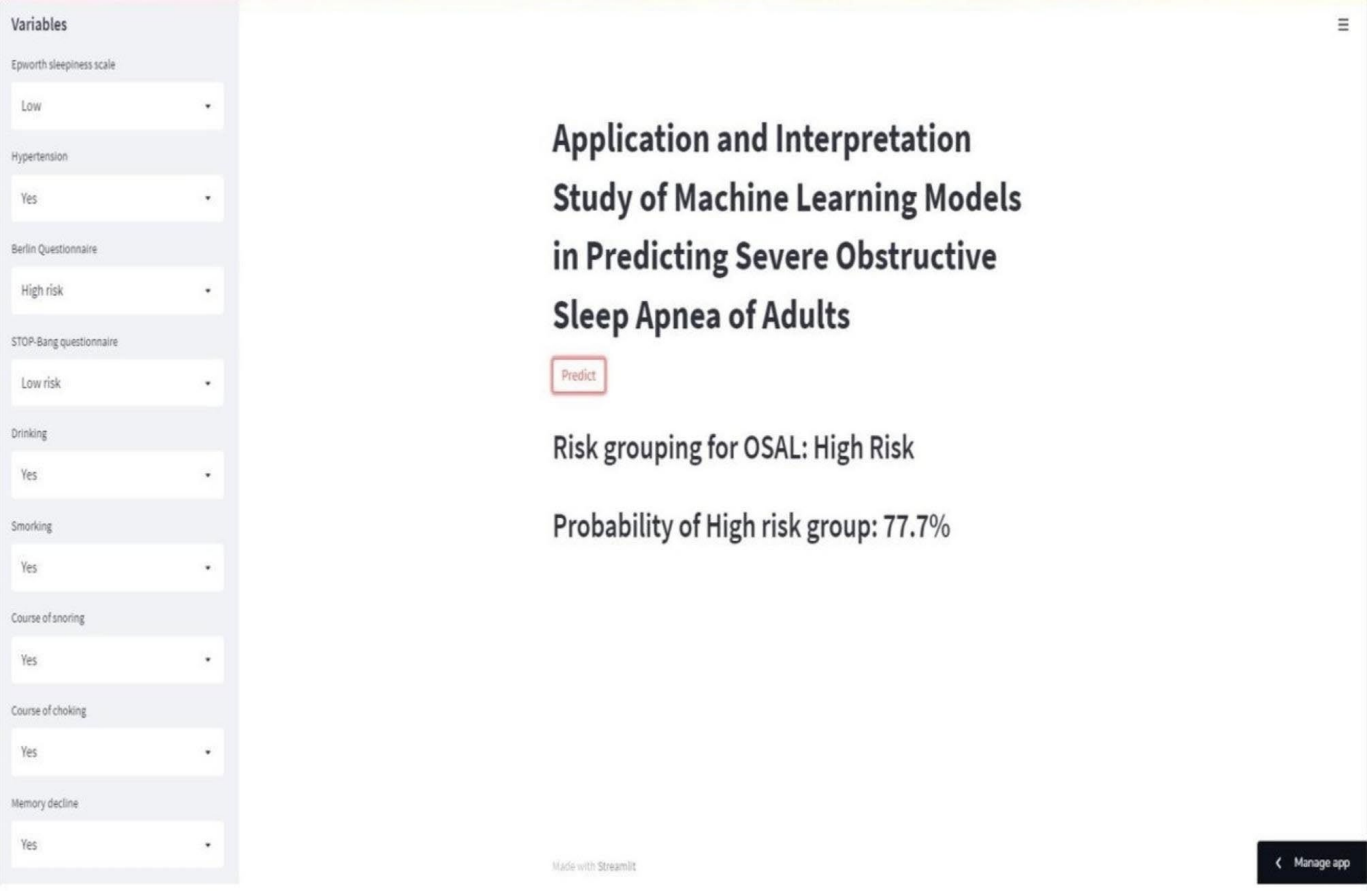
Online calculator predicting severe OSA in adults

An online calculator (https://hypertension-in--patients-with-osa-pmgz9gtjaaqzp4bi7iewp3.streamlit.app/) was developed based on the GBM model (Fig. 6). The risk of severe OSA is calculated by entering simple information. This can help clinicians treat patients with suspected severe OSA.

## Discussion

In the present study, we propose an interpretable ML model for predicting the risk of severe OSA. We used clinical features and sleep questionnaires from 1656 individuals for modeling, and then compared the prediction performance of six advanced ML models (LR, GBM, MLP, Bagging, AdaBoost, and XGBoost). Moreover, we analyzed the relative importance of risk factors by SHAP plots and interpreted the best model at the individual level through two typical cases. This demonstrated the application of a realistic database and interpretable ML method to develop a physician-understandable risk prediction model for severe OSA. This ML-based model facilitates early risk stratification and intervention for suspected OSA patients, making individualized medicine possible.

Previous studies on OSA prediction had two main characteristics: predicting the risk of OSA in the general population and selecting multiple types of input variables. Holfinger et al. utilized ML algorithms to make OSA prediction tools in clinical and community-based samples, and the ML models performed better than traditional logistic regression. It proved the feasibility of ML methods for OSA prediction [12]. Huo et al. made a ML derived questionnaire for screening OSA based on clinical information in two public datasets, and the model achieved AUC 0.78and 0.76, respectively [10]. Li et al. extracted features from multiple types of input signals, including oxygen saturation, electrocardiograms, airflow, thoracic, and abdominal signals. They used a multi-error-reduction classification system and gained the accuracy of 94.66% [41]. In the present study, we also considered these two aspects. When selecting the population and the target of prediction, we focused on people with suspected OSA symptoms who have a higher risk and severity of OSA. Due to the diverse symptoms of OSA, most patients are not diagnosed in time, leading to the detection of the disease in a severe stage [2]. Moreover, OSA can cause damage to multiple organ systems in several ways [4]. Common complications include hypertension, arrhythmia, cognitive disorders, and diabetes. Considering the importance and urgency, we selected the population with suspected OSA symptoms to predict severe OSA, a group with higher prevalence, complications and mortality. When selecting the input variables, the primary considerations were scientific rationality and easy accessibility. Therefore, we selected many questions that could be answered by the participants themselves. The questions were determined by experts based on the literature and their knowledge.

A total of 23 variables were collected in this study. After univariate analysis, 15 variables were selected for further model construction. The multivariate LR analysis showed that 10 variables were independent predictors of severe OSA. These variables could be classified into general characteristics (gender and age), body size parameters (BMI and waist circumference), lifestyle habits (smoking), sleep symptoms (snoring and breathing cessations during sleep), and sleep questionnaires suggesting a high risk of OSA (ESS and SBQ). This was generally consistent with previous studies. OSA was more common in men with high BMI and with increasing age [24]. The association could be explained through multiple mechanisms, including increased upper airway collapsibility and impaired neuromuscular control of upper airway patency due to local fat deposition. Waist circumference was also an important risk factor and predictor of OSA, along with BMI, and was significantly correlated with OSA severity [25]. Adverse lifestyles could also have a role in the rising prevalence of OSA. Smoking-related airway inflammation and disease may increase susceptibility to OSA [42]. Snoring, one of the most typical clinical symptoms of OSA, is very important in OSA diagnosis [43]. Breathing cessations during sleep indicate the severity of OSA. ESS and SBQ are used to evaluate the degree of daytime sleepiness and the risk of OSA, respectively; they are widely used for OSA screening [44].

We established six advanced ML models to predict the risk of severe OSA. The performance of models was ranked based on the AUC as follows: GBM (0.857) > XGBoost (0.840) > AdaBoost (0.825) > Bagging (0.814) > MLP (0.773) > LR (0.765). The GBM classification model showed the best performance with an accuracy of 0.766, a sensitivity of 0.798, and a specificity of 0.734. These ML models showed satisfactory performance, confirming the efficacy and applicability of ML in assessing the risk of severe OSA. In addition, most of the models tended to have higher sensitivity than specificity. In clinical practice, it is more desirable for predictive models to have better sensitivity to detect the disease in time. Moreover, the GBM model we proposed has advantages over similar models in previous studies in terms of number of participants and prediction performance. Huang et al. proposed a predictive model for severe OSA based on a support vector machine, which showed AUC, sensitivity, and specificity values of 0.780, 0.702, and 0.703, respectively [11]. He et al. established an LR model to determine the presence and severity of OSA. This model had a satisfactory performance in predicting the presence of OSA with an accuracy of 0.812. However, the accuracy was only 0.416 in predicting the severity of OSA [45]. Liu et al. established a support vector machine model to predict severe OSA; in males and females, the AUC was 0.765 and 0.830, respectively [46]. Laharnar et al. developed a scoring system for predicting severe OSA with an AUC of 0.900 and an accuracy, sensitivity, and specificity of 0.820. This system showed excellent performance but suffered from a small sample size and a low number of variables [47]. Zhang et al. developed a ML model to screen moderate to severe OSA based on the Chinese population faciocervical and anthropometric measurements. This model gained an AUC of 0.824, and was especially suitable for people without significant daytime sleepiness [48]. Kuan et al. proposed OSA predictions using age, sex and BMI incorporating ML algorithms. The validation AUCs of the two models in large populations were 0.806 and 0.807, respectively [49]. To better use the model, we further built an online calculator estimating the probability of severe OSA. Using online and self-reported data to screen for disease risk can improve clinical efficiency by providing an automated screening strategy.

Interpretability is an integral part of medical ML models. The goal of interpretable models is to make decisions similar to human behavior by providing explanations [50]. The currently proposed measures for explanation mainly analyze the model after training, i.e., post hoc interpretability [51]. We further used SHAP values to interpret the model. As shown in the SHAP summary plot, waist circumference, neck circumference, ESS, age, and BQ were among the most important prediction factors. At the individual level, high waist circumference, neck circumference, and BMI, smoking, breathing cessations during sleep, and a high-risk level of SBQ and BQ were risk factors of severe OSA; in contrast, low waist circumference, low neck circumference, and low ESS were protective factors. These results were in line with the multivariate LR analysis and previous findings. These results suggest that in addition to symptoms, we need to pay attention to the body size of patients in clinical work. In the case of insufficient diagnostic conditions, the scales have an important auxiliary judgment value. Weight loss may also be important for OSA patients.

Compared with previous studies, the present study is significant in terms of the research process and results. Unlike previous cases based only on public databases, we recruited subjects from northwestern China and collected their clinical information. Therefore, this study reflects the real situation of severe OSA patients, improving its applicability. Moreover, the included variables were all self-reported by subjects, facilitating generalization. In addition, previous studies only predicted OSA using one model, whereas we applied and compared various advanced ML methods and conducted an interpretability analysis of the most appropriate ML methods. Our proposed simplified model provides a screening platform for severe OSA, facilitates individualized risk stratification and formulates diagnostic decisions. It will help to identify patients with severe OSA as early as possible and provide timely treatment, especially in economically underdeveloped areas. Meanwhile, this model explains the importance of variables, which helps improve insights into severe OSA risk factors for clinicians.

There were several limitations in this study. First, the model we established was developed to detect severe OSA, and thus subjects with suspected OSA in whom the prevalence of OSA was high were enrolled. Therefore, the results may not apply to the general population, where the prevalence of OSA is much lower. Second, the prevalence of OSA varies greatly among regions and ethnicities [52]. This study was a single-center study, and all subjects were Chinese, so this model needs to be validated in populations of multiple ethnicities. Third, this was a retrospective study without follow-up data. Further prospective validation studies should include larger samples to obtain more reliable results. Fourth, the accuracy and stability of the model need to be evaluated and improved. In summary, the model must be improved before being generalized by increasing the sample size, adding more relevant variables, and optimizing the algorithm.

## Conclusions

In the present study, we developed six types of ML models to predict the risk of severe OSA, namely LR, MLP, Bagging, GBM, AdaBoost, and XGBoost. All six models showed high prediction performance. The GBM model performed best. Furthermore, we interpreted the model at the domain, feature, and individual levels. The findings of this study demonstrate the potential of a personalized screening model for individuals with a high risk of severe OSA using self-reported information. This will help to identify patients with severe OSA as early as possible and provide timely treatment, especially in economically underdeveloped areas. In the future, ML models are expected to make greater contributions to disease screening.

## Data Availability

Research data are not publicly available but can be obtained from the corresponding author on request after approval from the institutional review boards of all participating institutions.

## Abbreviations

AASM: American Academy of Sleep Medicine
AdaBoost: Adaptive boosting
AHI: Apnea-hypopnea index
AUC: Area under the receiver operating characteristic curve
Bagging: Bootstrapped aggregating
BMI: Body mass index
BQ: Berlin questionnaire
CI: Confidence intervals
ESS: Epworth sleepiness scale
GBM: Gradient boosting machine
IQR: Interquartile range
LR: Logistic regression
ML: Machine learning
MLP: Multilayer perceptron
OR: Odds ratio
OSA: Obstructive sleep apnea
PSG: Polysomnography
ROC: Receiver operating characteristic
SBQ: STOP-BANG questionnaire
SHAP: SHapley Additive exPlanations
XGBoost: Extreme gradient boosting
